# Changes in flavor substances during the processing of boneless cold‐eating rabbit meat

**DOI:** 10.1002/fsn3.3600

**Published:** 2023-08-06

**Authors:** Xianling Yuan, Xianjie Peng, Yidan Zheng, Yi Luo, Hongbin Lin, Zhouyou Zhang

**Affiliations:** ^1^ College of Bioengineering Sichuan University of Science and Engineering Yibin China; ^2^ Sichuan Swellfun Co., Ltd. Chengdu China; ^3^ Changning County Product Quality Inspection and Testing Center Yibin China; ^4^ Xihua University Chengdu China

**Keywords:** fatty acids, free amino acids, nucleotides, taste substance, volatile flavor substance

## Abstract

Cold‐eating rabbit is a traditional Chinese delicacy made by the process of pickling and frying. To explore the relationship between the flavor of cold‐eating rabbit and the production process, this study investigated the changes of nucleotides, free amino acids, fatty acids, and volatile flavor substances in diced, marinated for 10 min, marinated for 20 min, fried for 5 min, re‐fried for 10 min, re‐fried for 15 min, re‐fried for 20 min, seasoned and fried, and in the finished product, and analyzed the changes of flavor substances in deboned rabbit at different processing stages. Results showed that the content of 5′‐inosine monophosphate (IMP) increased significantly (*p* < .05), indicating that the degradation pathway mainly involved IMP. In total, 17 free amino acids were detected, the contents of which increased significantly (*p* < .05). In addition, 27 medium‐ and long‐chain fatty acids were detected. The total concentration of free fatty acids decreased in the fresh rabbit meat‐marinated 20 min stage (*p* < .05), then increased in the fried 5 min–fried 20 min stage (*p* < .05), and finally decreased in the fried with spices–finished stage (*p* < .05). Seventy‐seven volatile flavor substances were detected, and the 15‐minute frying stage was key in producing the volatile flavor substances.

## Graphical abstract

A total of 77 volatile flavors was detected. The types of volatile flavor substances were mainly alcohols, esters, and hydrocarbons that were transformed gradually into alcohols, acids, and others. Esters and acid compounds were the main flavor substances during the entire processing of cold‐eating rabbits. The 15 min stage of frying was the pivotal point for boneless, cold‐eating rabbits to produce volatile flavor substances.

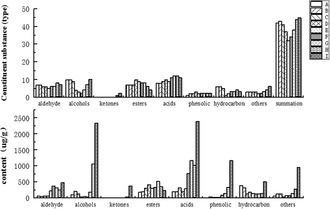

## 1 INTRODUCTION

Rabbit meat, high in protein, lysine, and lecithin, is easily digestible and is low in calories, uric acid, fat, and cholesterol (Cullere & Dalle Zotte, 2018), and is a fast‐growing efficient food converter (Lytle et al., 2021). It is also rich in selenium and phosphorus, and is an important source of vitamin B, which possesses anti‐hypertensive function (Stachniuk et al., 2020); in addition, it has the reputation of being a “beauty and health care product” among meats (Wang et al., 2021). Cold‐eating rabbit meat is a well‐developed product in the rabbit meat industry. Known for its spicy and delicious taste, it is popular with diners and has a promising future (Jian & Qiying, 2022).

Raw rabbit meat has a weak aroma, which attains flavor only when heated. Most of the flavor precursors are degraded when the reaction temperature is high; however, at the same time, a large number of reaction intermediates are produced, which are called primary degradation products. As the reaction continues, a large quantity of aromatic products characteristic of this meat are produced (Yi, 2015).

Cold‐eating rabbit meat undergoes a series of complex reactions during processing that produces precursors, intermediate reaction products, and degradation products (Aaslyng & Meinert, 2017), which, in turn, affect the flavor substances and volatile flavor substances of the product to generate unique flavors (Li et al., 2018). During the processing of cold‐eating rabbit meat, marination and frying are the key factors that affect the flavor of the product. Currently, the flavor substances of cold‐eating rabbit remain unclear. In this study, we aimed to analyze the changes in the levels of nucleotides, fatty acids, free amino acids, and flavor substances at each processing step and provide a theoretical basis for improving the flavor of cold‐eating rabbit meat. Our observations aim to lay the foundation for industrialization and standardization of cold‐eating rabbit industry.

## 2 MATERIALS AND METHODS

### 2.1 Sample preparation

**FIGURE 1:**
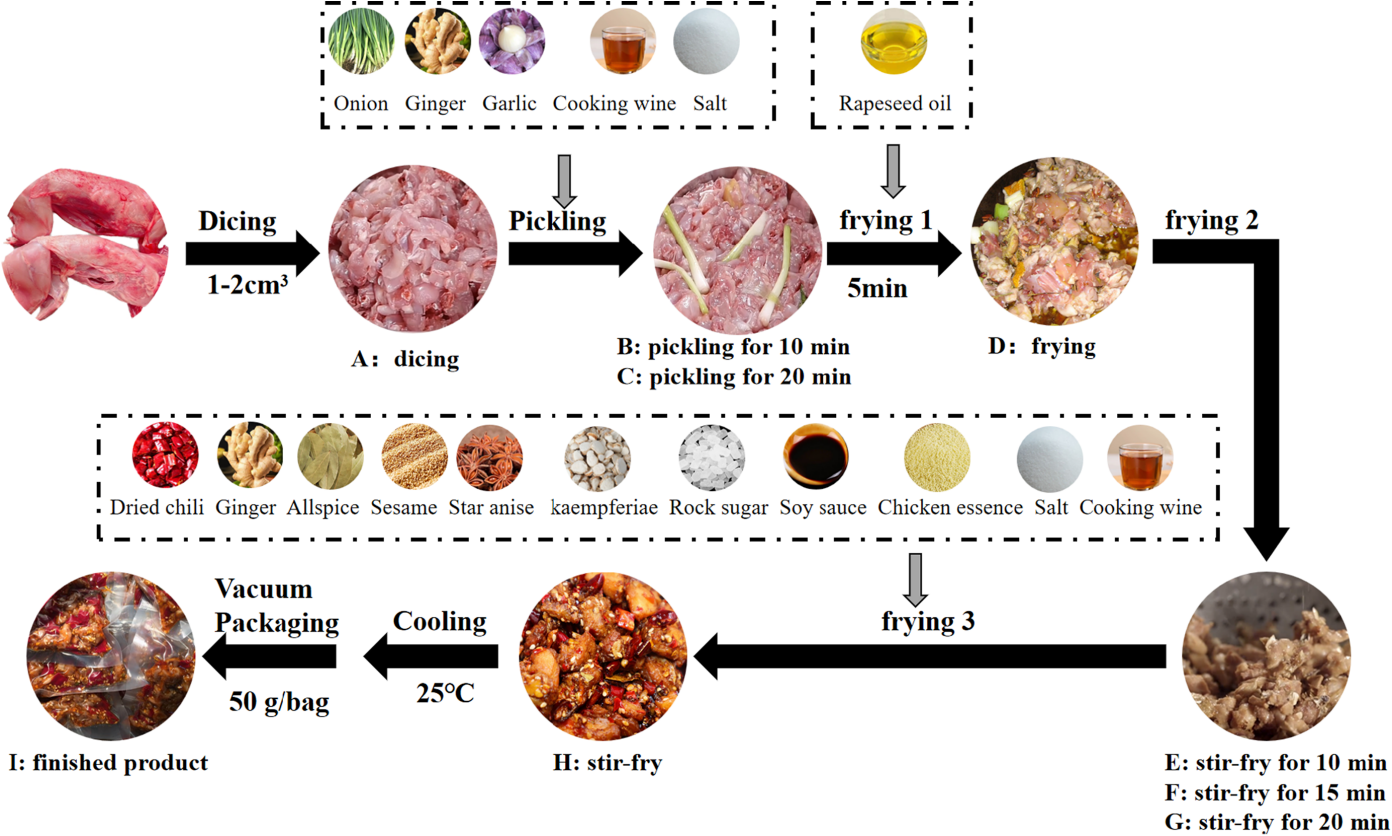
The production process and sampling points of cold rabbit meat. A: fresh rabbit meat; B: marinated for 10 min; C: marinated for 20 min; D: fried for 5 min; E: stir‐fried for 10 min; F: stir‐fried for 15 min; G: fried for 20 min; H: stir‐fried with spices; I: the finished product.

The production process of cold‐eating rabbit meat and the sampling points (A–I) are shown in Figure 1. At each sampling point, 30 g of sample was collected in sterile bags for each indicator, and each test was repeated three times.

### 2.2 Determination of physical and chemical composition

The moisture content in the samples was determined using the “direct drying method” in GB/T 5009.3‐2016 (SAC, 2016a), and an electric blast drying oven (101‐3AB, Zhongxing) was used to dry the samples in a constant weight. The protein content in the samples was determined using the “Kjeldahl method” in GB/T 5009.5‐2016 (SAC, 2016b), and an automatic Kjeldahl analyzer (kjeltec 8400, Foss Denmark) was used after digestion. The fat content of the samples was determined by referring to GB/T5009.6‐2016 (SAC, 2016c) using a soxlet extractor (SoxTecTM 2055 type, Foss China). Chloride content was determined following the “Silver Method” in GB/T 5009.44‐2016 (SAC, 2016d). pH was determined following the method used by Xiao et al. (2023) using an automatic potentiometric titrator (ZDJ‐4A, Lei Magnetic). Each sample was measured thrice to obtain an average value.

The fat content of the sample is calculated according to Equation (1).

(1) $$X=\frac{m_{1}-m_{0}}{m_{2}}\times 100$$

*X* is the content of fat in the specimen in grams per hundred grams (g/100 g), *m*_1_ is the content of the receiving bottle and fat in grams (g) after constant weight, *m*_0_ is the mass of the receiving bottle in grams (g), *m*_2_ is the mass of the specimen in grams (g), and 100 is the conversion factor.

### 2.3 Determination of nucleotide content

Ten grams of mashed, boneless, cold‐eating rabbit meat was homogenized in 50 mL 5% HClO_4_ for 30 s (24,200 *g*) and centrifuged at 4°C for 10 min (24,200 *g*), and the procedure was repeated once. The supernatant was combined and filtered using a medium‐speed filter paper. The pH of the filtered supernatant was adjusted to 6.0 using 1 and 0.5 M NaOH and set aside after passing through a 0.22 μm filter membrane. The sample solution was detected using high‐performance liquid chromatography (HPLC; 1260 Infinity II; Agilent). The parameters were set as follows: chromatographic column, Polaris 5 C18‐A, 250 × 4.6 nm; detection wavelength, 254 nm; column temperature, 30°C; mobile phase A, 0.05 M K_2_HPO_4_; phase B, methanol of HPLC grade; A:B = 95:5 of equal gradient washing; flow rate, 0.8 mL/min; and injection volume, 20 μm (Duan et al., 2020).

### 2.4 Determination of free amino acid content

According to the “Determination of Amino Acids in Foods” in GB/T 5009.124‐2016 (SAC, 2016e), 0.1 g boneless cold‐eating rabbit meat was weighed into a hydrolysis tube that contained 10 mL 6 M hydrochloric acid, to which three drops of phenol were added. After filling with nitrogen, the mixture was vacuumed. This step was repeated three times, after which it was sealed. The hydrolysis tube was put in an electric heating blast drying box for hydrolysis at 110°C for 24 h. Then, it was taken out and cooled to room temperature. After opening the hydrolysis tube, the mixture was filtered with medium‐speed filter paper and mixed with distilled water for dilution in a 100 mL volumetric flask. Then, 200 μL of the solution was taken and dried under reduced pressure in a drying box. Next, 200 μL sodium citrate (pH 2.22) was added, passed through a 0.22 μm filter membrane, and detected using HPLC (Hitachi, L‐8900).

### 2.5 Determination of fatty acid content

According to the “internal standard method” in GB/T 5009.168‐2016 (SAC, 2016f), 5 g sample, 2 mL of 95% ethanol, 4 mL ultrapure water, and 10 mL of 8.3 mol/L hydrochloric acid solution were weighed and mixed well. The colorimetric tubes were placed in a water bath at 80°C for 40 min, with shaking every 10 min. The tube for color comparison was removed and cooled to room temperature, followed by the addition of 10 mL of 95% ethanol and mixing. Next, 100 mL of a 1:1 mixture of petroleum ether: ether was added for extraction; the extraction was performed thrice and the petroleum ether: ether was dried using steam in a water bath. Then, 4 mL of sodium hydroxide–methanol solution was added to the fat extract, incubated in a water bath at 45°C for 20 min, and cooled to room temperature, followed by the addition of 4 mL boron trifluoride‐methanol solution and incubation in a water bath at 45°C for 20 min. Finally, 3 mL of n‐hexane was added as a layer at room temperature for extraction and then n‐hexane layer through the film before machine.

For chromatography, an HP‐88 Agilent column was used. The flame ionization detector (FID) temperature was set at 280°C and the inlet temperature at 250°C, and the injection split ratio was 20:1. The N_2_ carrier gas flow rate was set at 1.0 mL/min. The heating procedure included incubation at an initial temperature of 100°C for 13 min, which was increased to 180°C at 10°C/min, maintained for 6 min, then raised to 192°C at the rate of 1.5°C/min, maintained for 6 min, and then heated to 240°C at the rate of 3.5°C/min and maintained for 4 min.

### 2.6 Determination of volatile flavor substances

Using the method of headspace solid‐phase microextraction (Guo et al., 2020), 4 g boneless cold‐eating rabbit meat was mashed and put into a headspace injection bottle (20 mL, Supelco). The aged extraction tip (65 μm PDMS/DVB, Supelco) was inserted into the sample vial, equilibrated at 50°C for 30 min, and then placed in the syringe port and resolved at 200°C for 5 min. Then, the extracted solution was analyzed using a gas chromatography‐mass spectrometer under chromatographic conditions. The detection conditions were as follows: inlet temperature was 200°C with splitless injection, the chromatographic column was DP‐WAX, the carrier gas flow rate was 1 mL/min, the initial temperature of the column oven was 40°C, and the final temperature was 200°C. For mass spectrometry, the ion source temperature was set at 280°C and the transmission line temperature at 260°C, and the scanning range was 30–550 *m*/*z*. Qualitative and quantitative analyses were performed as follows:

*Qualitative analysis*: The mass spectra of the compounds were identified in Puku (NIST11, NIST11s), and compounds with match degree >80 were recorded.
*Quantitative analysis*: Forty microliters of 0.2 μg/mL of 2‐ethylbutyric acid was added as an internal standard before headspace solid‐phase microextraction, and the relative concentration of each compound was calculated via area normalization.

### 2.7 Statistical analysis

The experimental results were statistically analyzed using Excel 2013. Analysis of variance (ANOVA) was performed for each index using SPSS, and the significance of differences was analyzed using the LSD method in the multiple comparison method, with *p* < .05 indicating significant differences (Xun et al., 2020). The Origin 2018 software was used for plotting.

## 3 RESULTS AND DISCUSSION

### 3.1 Analysis of the physical and chemical components during the processing of boneless cold‐eating rabbit meat

Moisture content differed significantly during stages A–H (*p* < .05; Table 1). Water evaporated from the meat during heating, which resulted in significant reduction in water content; however, significant differences were not observed during stages H‐I (*p* > .05). The percentage of fat in the samples first increased and then decreased because heating reduced the moisture content, which increased the percentage of fat (*p* < .05). However, after stage F, the fat percentage reduced significantly because of the enhanced thermal degradation of fat and production of fatty acids (*p* < .05; Wang, 2015). During the curing process, chloride concentration in meat increased significantly because of the osmotic effect, although the concentration decreased gradually during the frying process; possibly, fat inhibited the release of chloride and reduced chloride concentration (Chabanet et al., 2013). After stage H, the chloride concentration increased again after adding soy sauce and other seasonings. After marinating, the pH was significantly lower in the treated meat than in fresh rabbit meat (*p* < .05), although the pH increased after frying and then began to decrease.

**TABLE 1:** Changes in physical and chemical components in the processing of boneless, cold‐eating rabbits.

Indicators (%)	Different processing stages								
	A	B	C	D	E	F	G	H	I
Moisture	76.01 ± 1.24^a^	74.03 ± 0.08^b^	70.95 ± 0.50^c^	56.52 ± 1.30^d^	50.61 ± 0.70^e^	31.24 ± 0.36^f^	25.34 ± 0.93^g^	24.16 ± 0.20^h^	23.02 ± 0.06^h^
Protein	37.61 ± 0.34^d^	36.17 ± 0.18^e^	33.95 ± 0.20^f^	30.44 ± 0.06^e^	23.66 ± 0.34^e^	23.85 ± 0.40^c^	27.63 ± 0.12^a^	26.01 ± 0.12^b^	27.68 ± 0.30^a^
Fat	2.61 ± 0.01^i^	8.58 ± 0.18^h^	10.22 ± 0.10^g^	20.47 ± 0.17^f^	22.14 ± 0.04^e^	37.03 ± 0.17^a^	28.44 ± 0.18^b^	27.21 ± 0.28^c^	25.65 ± 0.14^d^
Chloride	0.19 ± 0.02^e^	0.50 ± 0.03^d^	0.64 ± 0.04^c^	0.69 ± 0.03^c^	0.71 ± 0.02^c^	0.62 ± 0.04^c^	0.58 ± 0.03^c^	1.03 ± 0.05^a^	0.95 ± 0.03^b^
pH	5.94 ± 0.01^d^	5.66 ± 0.02^e^	5.62 ± 0.05^f^	6.08 ± 0.01^ab^	6.06 ± 0.02^abc^	6.05 ± 0.02^bc^	6.03 ± 0.03^c^	6.07 ± 0.01^abc^	6.09 ± 0.02^a^

*Note*: Different lowercase letters in the same line indicate significant difference (*p* < .05); A: fresh rabbit meat; B: marinate for 10 min; C: marinate for 20 min; D: fry for 5 min; E: stir‐frying for 10 min; F: stir‐frying for 15 min; G: fry for 20 min; H: stir‐fry with spices; I: the finished product.

### 3.2 Nucleotide analysis during processing of boneless cold‐eating rabbit meat

ATP and its related products, such as adenosine triphosphate (ATP), adenosine diphosphate (ADP), adenosine monophosphate (AMP), inosine monophosphate (IMP), hypoxanthine riboside (HxR), hypoxanthine (Hx), and adenosine riboside (AdR), are important components of boneless cold‐eating rabbit meat. ADP and AMP are produced from ATP via two degradation pathways: ATP → ADP → AMP → AdR → HxR → Hx → Xt or ATP → ADP → AMP → IMP→ HxR → Hx → Xt (Gu et al., 2020). The nucleotides, 5′‐guanylic acid (5′‐GMP), 5′‐IMP, and 5′‐AMP are the main sources of umami taste (Sun et al., 2010), while Hx synergized with some amino acids or peptides to produce a bitter flavor (Hernández‐Cázares et al., 2011).

As the concentration of 5′‐IMP increased significantly from 22.36 mg/100 g in stage A to 37.79 mg/100 g in stage I (*p* < .05; Table 2), we inferred that the IMP pathway was the main degradation pathway utilized during the processing of boneless cold‐eating rabbit meat. This was similar to the results of a study on ATP degradation in shrimp meat during heating (Luhao et al., 2020). The threshold value of 5′‐IMP was 25 mg/100 g, which indicated that 5′‐IMP was the most flavor‐imparting nucleotide in the meat. It has been shown that 5′‐IMP was the main flavor compound in poultry, livestock, fish, and other meat, and that it played an important role in the formation of meat flavor (Ramalingam et al., 2019). The concentrations of 5′‐GMP, 5′‐AMP, and Hx increased significantly with processing (*p* < .05), and the changes were basically consistent with the reports on nucleotide content during the processing of spiced duck (Shuangjuan, 2012). On the contrary, the concentration of 5′‐ADP decreased significantly from 5.22 mg/100 g to 1.06 mg/100 g (*p* < .05) with progress in processing. This may have been due to the degradation of ATP into ADP, which was decomposed into AMP by phosphokinase; AMP was then deaminated by AMP deaminase to produce IMP, which was transformed slowly into HxR and Hx by phosphatase (Kavitha & Modi, 2007).

**TABLE 2:** Changes in nucleotides (dry base) during the processing of boneless, cold‐eating rabbits.

Nucleotide (mg/100 g)	Different processing stages								
	A	B	C	D	E	F	G	H	I
5′‐IMP	22.36 ± 0.46^e^	22.11 ± 0.39^e^	20.81 ± 0.32^f^	15.87 ± 0.39^g^	21.03 ± 0.94^f^	26.68 ± 0.49^c^	32.01 ± 0.32^b^	23.93 ± 0.26^d^	37.79 ± 0.75^a^
5′‐GMP	2.09 ± 0.07^d^	2.49 ± 0.02^b^	2.16 ± 0.04^cd^	1.32 ± 0.05^g^	1.47 ± 0.04^f^	1.95 ± 0.03^e^	2.23 ± 0.07^c^	2.00 ± 0.03^e^	2.59 ± 0.04^a^
5′‐AMP	16.17 ± 0.05^g^	12.43 ± 0.04^h^	12.67 ± 0.02^h^	17.76 ± 0.06^f^	23.87 ± 0.13^e^	29.44 ± 0.27^c^	42.47 ± 0.31^a^	25.75 ± 0.09^d^	35.58 ± 0.54^b^
Hx	1.59 ± 0.03^g^	1.30 ± 0.01^h^	1.35 ± 0.03^h^	3.08 ± 0.03^f^	3.69 ± 0.03^e^	4.16 ± 0.06^c^	4.99 ± 0.04^b^	3.97 ± 0.05^d^	6.14 ± 0.03^a^
5′‐ADP	5.22 ± 0.03^a^	4.27 ± 0.01^b^	4.34 ± 0.02^b^	2.88 ± 0.03^c^	2.18 ± 0.02^e^	2.44 ± 0.08^d^	2.62 ± 0.04^cd^	2.41 ± 0.03^d^	1.06 ± 0.05^f^
Umami nucleotides	40.62 ± 2.36^e^	37.03 ± 1.61^f^	35.01 ± 2.14^g^	34.95 ± 3.77^g^	46.37 ± 3.26^d^	58.07 ± 1.94^b^	76.71 ± 1.06^a^	51.68 ± 2.14^c^	75.96 ± 1.58^a^

*Note*: Different lowercase letters in the same line indicate significant difference (*p* < .05); A: fresh rabbit meat; B: marinated for 10 min; C: marinated for 20 min; D: fried for 5 min; E: stir‐fried for 10 min; F: stir‐dried for 15 min; G: fried for 20 min; H: stir‐fried with spices; I: the finished product.

### 3.3 Analysis of free amino acids during the processing of boneless cold‐eating rabbit meat

During the heating of meat or meat products, protein is degraded into free amino acids, low‐molecular‐weight peptides, aldehydes, or organic acids, because of which the concentration of free amino acids increases continuously (Leggio et al., 2012). The amino groups of some free amino acids undergo the Maillard reaction with carbonyl groups that reduces sugars to produce a series of flavor substances (Geng et al., 2019). Based on the analysis of the chemical composition of meat, we speculated that free amino acids play an important role in the taste of meat (Rikimaru & Takahashi, 2010). According to the taste characteristics, free amino acids are divided into three categories: umami‐free amino acids (UFAA), sweet‐free amino acids (SFAA), and bitter‐free amino acids (BFAA).

In total, 17 types of amino acids were detected in cold‐eating rabbit meat, which included seven essential amino acids (Table 3). There was a significant upward trend during processing (*p* < .05), from 320.82 to 748.49 mg/100 g. The concentrations of UFAA, SFAA, and BFAA increased by 208.02 mg/100 g, 89.94 mg/100 g, and 70.22 mg/100 g, respectively, which was due to the decomposition of protein in rabbit meat during processing. However, the concentrations in stages B and C and in stages E and F did not change significantly (*p* > .05). In stages G–I, the content increased slowly, which may have been caused by the Maillard reaction that produced small molecule flavor substances, such as ketones, acids, and aldehydes. UFAA accounted for 38.46%–53.36% of the TFAA concentration, which was more than those of SFAA and BFAA, indicating that the umami taste is stronger than the sweet and bitter tastes.

**TABLE 3:** Changes of free amino acids (dry base) in boneless, cold‐eating rabbits during processing.

FAAs (mg/100 g)	Different processing stages								
	A	B	C	D	E	F	G	H	I
Asp	17.15 ± 0.08^g^	18.63 ± 0.20^f^	18.85 ± 0.08^f^	21.36 ± 0.57^e^	22.43 ± 0.26^d^	22.67 ± 0.08^d^	37.39 ± 0.24^c^	29.88 ± 0.15^b^	46.56 ± 0.20^a^
Glu	129.24 ± 0.27^h^	131.95 ± 0.09^g^	133.09 ± 0.10^f^	136.10 ± 0.71^e^	238.64 ± 0.11^d^	238.98 ± 0.13^d^	261.66 ± 0.61^c^	265.48 ± 0.57^b^	307.85 ± 0.65^a^
**UFAA**	**146.39 ± 1.27** ^**g**^	**150.58 ± 0.98** ^**f**^	**151.94 ± 0.73** ^**f**^	**157.46 ± 1.54** ^**e**^	**261.07 ± 0.38** ^**d**^	**261.65 ± 1.01** ^**d**^	**299.05 ± 0.21** ^**b**^	**295.36 ± 0.14** ^**c**^	**354.41 ± 0.75** ^**a**^
Thr	8.42 ± 0.06^h^	9.00 ± 0.03^g^	9.28 ± 0.02^f^	10.59 ± 0.08^e^	11.28 ± 0.11^d^	11.41 ± 0.06^d^	18.30 ± 0.21^c^	19.59 ± 0.17^b^	20.73 ± 0.12^a^
Ser	7.08 ± 0.04^i^	7.64 ± 0.06^h^	7.90 ± 0.04^g^	9.18 ± 0.05^f^	9.64 ± 0.07^e^	9.83 ± 0.07^d^	15.42 ± 0.12^c^	16.12 ± 0.05^b^	17.84 ± 0.19^a^
Gly	7.75 ± 0.09^g^	8.22 ± 0.20^f^	9.20 ± 0.15^e^	12.23 ± 0.07^c^	11.27 ± 0.21^d^	11.59 ± 0.16^d^	17.36 ± 0.44^b^	17.45 ± 0.23^b^	18.71 ± 0.16^a^
Ala	10.49 ± 0.25^f^	11.11 ± 0.51^e^	11.87 ± 0.15^d^	13.87 ± 0.13^c^	13.79 ± 0.17^c^	14.05 ± 0.06^c^	24.04 ± 0.11^b^	24.45 ± 0.12^b^	25.19 ± 0.55^a^
Pro	28.07 ± 0.42^h^	36.56 ± 0.17^e^	29.52 ± 0.46^g^	46.48 ± 0.34^c^	35.95 ± 0.27^f^	38.29 ± 0.11^d^	54.85 ± 0.19^b^	55.00 ± 0.17^b^	57.14 ± 0.04^a^
Lys	23.08 ± 0.32^h^	28.65 ± 0.11^g^	30.05 ± 0.31^f^	44.62 ± 0.48^c^	34.75 ± 0.38^d^	32.48 ± 0.35^e^	48.64 ± 0.40^a^	45.76 ± 0.17^b^	44.86 ± 0.32^c^
Val	9.67 ± 0.31^f^	9.90 ± 0.18^f^	10.32 ± 0.23^f^	11.48 ± 0.33^e^	12.32 ± 0.38^d^	12.50 ± 0.14^d^	20.53 ± 0.22^c^	22.48 ± 0.54^b^	23.60 ± 0.47^a^
**SFAA**	**94.56 ± 0.72** ^**g**^	**111.08 ± 0.58** ^**e**^	**108.14 ± 1.31** ^**f**^	**148.45 ± 0.54** ^**c**^	**129.00 ± 0.91** ^**d**^	**130.15 ± 0.58** ^**d**^	**199.14 ± 0.32** ^**b**^	**200.85 ± 0.16** ^**b**^	**184.50 ± 0.85** ^**a**^
Cys	0.89 ± 0.04^c^	1.02 ± 0.03^b^	1.04 ± 0.06^b^	0.06 ± 0.06^d^	0.93 ± 0.08^bc^	0.84 ± 0.09^c^	0.02 ± 0.02^d^	1.37 ± 0.07^a^	1.37 ± 0.02^a^
Met	1.73 ± 0.15^h^	5.07 ± 0.08^g^	5.45 ± 0.05^f^	5.82 ± 0.11^e^	6.22 ± 0.26^d^	6.39 ± 0.07^d^	11.50 ± 0.24^c^	12.20 ± 0.16^b^	12.53 ± 0.10^a^
Leu	15.94 ± 0.24^g^	16.49 ± 0.12^f^	16.71 ± 0.21^f^	18.86 ± 0.21^e^	20.57 ± 0.19^d^	20.84 ± 0.24^d^	34.00 ± 0.18^c^	36.39 ± 0.22^b^	36.53 ± 0.51^a^
Arg	11.06 ± 0.08^i^	11.86 ± 0.15^h^	12.83 ± 0.09^g^	14.97 ± 0.14^f^	15.43 ± 0.12^e^	15.77 ± 0.09^d^	25.12 ± 0.30^c^	27.18 ± 0.16^b^	28.80 ± 0.34^a^
Ile	8.61 ± 0.49^f^	9.12 ± 0.19^f^	9.12 ± 0.27^f^	10.74 ± 0.10^e^	11.28 ± 0.11^de^	11.72 ± 0.29^d^	18.91 ± 0.20^c^	20.46 ± 0.35^b^	20.54 ± 0.80^a^
Tyr	7.82 ± 0.12^h^	7.50 ± 0.09^h^	8.17 ± 0.21^g^	9.12 ± 0.11^f^	9.82 ± 0.08^e^	10.27 ± 0.33^d^	16.22 ± 0.31^c^	17.11 ± 0.11^b^	17.29 ± 0.15^a^
Phe	7.29 ± 0.32^e^	6.16 ± 0.18^f^	6.00 ± 0.21^f^	8.26 ± 0.35^d^	7.77 ± 0.27^de^	8.25 ± 0.12^d^	14.41 ± 0.44^c^	15.50 ± 0.48^b^	16.27 ± 0.18^a^
His	26.53 ± 0.30^b^	20.86 ± 0.33^e^	16.44 ± 0.17^f^	35.65 ± 0.43^a^	27.50 ± 0.19^b^	24.47 ± 0.56^d^	26.64 ± 1.08^b^	25.30 ± 0.26^c^	16.86 ± 0.28^f^
**BFAA**	**79.87 ± 0.41** ^**f**^	**78.08 ± 0.97** ^**f**^	**75.76 ± 0.59** ^**g**^	**103.48 ± 0.61** ^**d**^	**99.52 ± 1.07** ^**e**^	**98.55 ± 0.55** ^**e**^	**146.82 ± 0.42** ^**c**^	**155.51 ± 0.26** ^**b**^	**150.09 ± 1.33** ^**a**^
**TFAA**	**320.82 ± 0.67** ^**g**^	**339.74 ± 0.03** ^**f**^	**336.84 ± 0.17** ^**f**^	**409.39 ± 0.56** ^**e**^	**489.59 ± 0.41** ^**d**^	**490.35 ± 0.82** ^**d**^	**645.01 ± 0.33** ^**c**^	**651.74 ± 0.06** ^**b**^	**689.00 ± 0.14** ^**a**^

*Note*: Different lowercase letters in the same line indicate significant difference (*p* < .05); A: fresh rabbit meat; B: marinated for 10 min; C: marinated for 20 min; D: fried for 5 min; E: stir‐fried for 10 min; F: stir‐fried for 15 min; G: fried for 20 min; H: stir‐fried with spices; I: the finished product.

Asp and Glu are UFAAs. The concentration of Glu increased the most, and combined with its threshold value of 30 mg/100 g, Glu contributed significantly to the umami taste in boneless cold‐eating rabbit meat; it also acted synergistically with chloride to produce the umami taste of MSG (Gunlu & Gunlu, 2014). This is similar to the results of Rikimaru and Takahashi (2010) who found high levels of Glu in chicken and shrimp paste (Zhu et al., 2019). SFAA mainly contains seven types of free amino acids. The concentrations of these also increased during processing. Among them, Pro content increased by 29.07 mg/100 g, followed by those of Lys and Ala, the contents of which increased by 21.78 mg/100 g and 14.70 mg/100 g, respectively. At present, Lys analogs are used in the treatment of prostaglandins (Fergusson et al., 2008). Ala not only has a sweetening effect, but its transaminated form can also be used to treat chronic hepatitis (Chien et al., 1999); this shows that cold‐eating rabbit is a healthy food. In BFAA, the concentration of Leu increased by 20.59 mg/100 g, followed by that of Arg, which increased to 17.74 mg/100 g.

### 3.4 Analysis of fatty acids during processing of boneless cold‐eating rabbit meat

During the heating process, high temperatures cause degradation of fatty acids. Several flavor compounds (Mottram, 1992) are generated via a thermal oxidation reaction at high temperature (Deng et al., 2015) that are more conducive to the formation of aldehydes or ketones, such as 2,4‐decadienal, 2‐nonenal, and 1‐octene‐3‐one (Shi et al., 2020). Fatty acids can be categorized into saturated fatty acids (SFA) and unsaturated fatty acids (UFA), and UFAs are divided further into monounsaturated fatty acids (MUFA) and polyunsaturated fatty acids (PUFA).

In total, 27 medium‐ and long‐chain fatty acids were detected (Table 4). Thirteen SFAs were present at concentrations of 340.44–1643.06 mg/100 g, six MUFAs were present at concentrations of 370.62–11158.1 mg/100 g, and eight PUFAs showed concentrations of 449.61–6103.35 mg/100 g. The concentrations of UFAs were higher than those of SFAs, which was similar to the results of Xun et al. (2020) who analyzed the concentrations of fatty acids in chicken. The concentrations of MUFAs were relatively higher than that of PUFAs. Xiaozhou (2020) also obtained similar results using the Yellow River carp.

**TABLE 4:** Changes of fatty acids in boneless, cold‐eating rabbits during processing (dry base).

FAs (mg/100 g)	Different processing stages								
	A	B	C	D	E	F	G	H	I
C10:0	2.87 ± 0.21^cd^	2.13 ± 0.15^f^	1.87 ± 0.06^f^	3.10 ± 0.2^c^	4.20 ± 0.1^b^	6.27 ± 0.06^a^	2.70 ± 0.10^d^	2.10 ± 0.1^f^	2.43 ± 0.15^c^
C12:0	3.27 ± 0.00^d^	2.47 ± 0.21^f^	1.97 ± 0.06^g^	3.33 ± 0.15^d^	5.03 ± 0.15^b^	6.83 ± 0.15^a^	4.47 ± 0.35^c^	3.57 ± 0.21^d^	2.87 ± 0.15^e^
C13:0	0.4 ± 0.00^b^	0.43 ± 0.06^b^	N	N	N	N	0.63 ± 0.06^a^	0.63 ± 0.06^a^	0.63 ± 0.06^a^
C14:0	47.00 ± 2.92^c^	35.97 ± 1.17^e^	24.53 ± 1.38^g^	39.93 ± 1.84^d^	63.87 ± 1.17^a^	66.37 ± 0.75^a^	64.20 ± 0.89^a^	54.10 ± 1.70^b^	28.67 **±** 1.32^f^
C15:0	12.37 ± 0.31^c^	10.93 ± 0.35^d^	8.17 ± 0.15^f^	12.33 ± 0.35^c^	18.77 ± 0.31^a^	19.40 ± 0.17^a^	19.23 ± 0.25^a^	15.63 ± 0.57^b^	9.47 ± 0.25^e^
C16:0	268.37 ± 9.42^f^	235.50 ± 12.36^g^	168.43 ± 12.8^h^	455.40 ± 13.17^d^	638.00 ± 5.12^b^	662.80 ± 31.04^b^	743.43 ± 12.81^a^	520.20 ± 25.52^c^	328.87 **±** 11.15^e^
C17:0	15.50 ± 0.66^c^	14.20 ± 0.78^c^	10.77 ± 0.75^d^	17.83 ± 1.55^b^	27.43 ± 1.04^a^	26.47 ± 0.49^a^	28.27 ± 1.46^a^	19.07 ± 1.17^b^	13.63 ± 0.60^c^
C18:0	167.0 ± 7.35^g^	158.3 ± 2.76^g^	117.8 ± 4.79^h^	336.9 ± 3.73^e^	441.7 ± 4.10^c^	487.5 ± 6.27^b^	563.2 ± 7.08^a^	381.8 ± 4.60^d^	260.6 ± 10.65^f^
C20:0	3.43 ± 0.07^f^	3.63 ± 0.07^f^	2.43 ± 0.32^f^	64.00 ± 2.55^d^	86.43 ± 0.91^c^	106.9 ± 8.81^b^	121.0 ± 3.91^a^	67.53 ± 2.89^d^	46.13 ± 3.13^e^
C21:0	0.44 ± 0.07^d^	0.52 ± 0.08^d^	0.98 ± 0.05^c^	1.93 ± 0.09^b^	2.73 ± 0.09^a^	N	N	1.90 ± 0.27^b^	1.10 ± 0.19^c^
C22:0	1.29 ± 0.15^f^	1.25 ± 0.14^f^	1.38 ± 0.20^f^	35.77 ± 2.32^d^	51.93 ± 2.44^c^	58.10 ± 1.95^b^	66.20 ± 2.33^a^	37.30 ± 2.63^d^	25.87 ± 2.41^e^
C23:0	0.79 ± 0.03^d^	0.89 ± 0.05^d^	0.68 ± 0.05^d^	2.41 ± 0.16^c^	4.33 ± 0.24^a^	N	N	2.73 ± 0.07^b^	N
C24:0	1.21 ± 0.06^f^	1.18 ± 0.02^f^	1.43 ± 0.17^f^	17.60 ± 2.10^d^	25.60 ± 1.37^c^	36.43 ± 0.80^a^	29.73 ± 1.31^b^	17.27 ± 1.15^d^	12.93 ± 1.25^e^
**SFAs**	**523.94 ± 0.63** ^**g**^	**467.40 ± 0.48** ^**h**^	**340.44 ± 0.11** ^**i**^	**990.53 ± 0.57** ^**e**^	**1370.02 ± 0.38** ^**c**^	**1477.07 ± 0.05** ^**b**^	**1643.06 ± 0.64** ^**a**^	**1123.83 ± 0.31** ^**d**^	**733.20 ± 0.52** ^**f**^
C14:1	2.07 ± 0.31^cd^	1.23 ± 0.12^e^	0.97 ± 0.15^e^	1.90 ± 0.17^d^	3.27 ± 0.21^b^	4.43 ± 0.25^a^	2.43 ± 0.15^c^	2.27 ± 0.21^cd^	1.20 ± 0.26^e^
C16:1	41.07 ± 2.45^e^	33.13 ± 3.17^f^	21.87 ± 2.15^g^	56.43 ± 1.43^d^	93.80 ± 3.84^a^	77.93 ± 3.02^c^	87.17 ± 3.37^b^	61.47 ± 3.51^d^	40.23 ± 3.84^e^
C18:1N9C	445.3 ± 10.5^f^	400.4 ± 12.8^f^	284.9 ± 10.9^f^	5825.9 ± 85.2^d^	8038.8 ± 162.2^c^	8867.0 ± 321.5^b^	10674.1 ± 300.0^a^	5867.7 ± 141.5^d^	3850.1 **±** 176.1^e^
C20:1	6.40 ± 0.38^e^	4.40 ± 0.63^e^	2.97 ± 0.20^e^	144.9 ± 11.70^c^	229.7 ± 18.80^b^	241.9 ± 15.86^b^	263.47 ± 13.43^a^	145.6 ± 7.89^c^	88.80 ± 5.50^d^
C22:1N9	56.50 ± 1.42^d^	58.00 ± 1.20^d^	58.73 ± 0.96^d^	74.73 ± 1.56^c^	75.13 ± 2.17^c^	80.40 ± 1.93^b^	95.73 ± 2.11^a^	98.41 ± 1.78^a^	77.13 ± 1.17^c^
C24:1	1.19 ± 0.16^f^	0.97 ± 0.08^f^	1.18 ± 0.09^f^	22.30 ± 1.45^d^	27.67 ± 1.44^c^	32.37 ± 1.21^b^	35.20 ± 0.95^a^	21.60 ± 1.51^d^	13.27 ± 1.12^e^
**MUFAs**	**552.53 ± 4.55** ^**f**^	**498.13 ± 2.62** ^**f**^	**370.62 ± 1.47** ^**g**^	**6126.16 ± 21.02** ^**d**^	**8467.96 ± 58.46** ^**c**^	**9304.03 ± 174.2** ^**b**^	**11158.1 ± 125.3** ^**a**^	**6197.05 ± 76.2** ^**d**^	**4070.73 ± 53.9** ^**e**^
C18:2N6C	593.8 ± 7.78^f^	563.3 ± 15.5^f^	409.7 ± 19.5^f^	2375.7 ± 78.6^d^	3280.1 ± 314.6^c^	3674.6 ± 262.6^b^	4193.8 ± 245.6^a^	2485.9 ± 178.0^d^	1547.1 ± 186.1^e^
C18:3N6	1.07 ± 0.13^d^	1.52 ± 0.21^d^	1.15 ± 0.08^d^	7.63 ± 0.31^c^	9.32 ± 0.33^b^	12.50 ± 0.98^a^	N	N	N
C18:3N3	34.60 ± 0.95^f^	37.43 ± 1.33^f^	24.33 ± 1.86^f^	975.1 ± 28.98^d^	1395.9 ± 41.8^c^	1557.2 ± 58.4^b^	1763.5 ± 70.8^a^	962.3 ± 40.1^d^	642.8 ± 17.2^e^
C20:2	7.66 ± 0.37^f^	6.50 ± 0.51^f^	4.85 ± 0.37^f^	13.20 ± 2.19^e^	21.23 ± 2.32^c^	24.43 ± 1.70^b^	27.10 ± 1.28^a^	18.33 ± 0.65^d^	12.37 ± 1.19^e^
C20:3N6	6.98 ± 0.13^d^	7.09 ± 0.14^d^	6.82 ± 0.07^d^	7.69 ± 0.14^d^	25.27 ± 1.93^a^	8.90 ± 0.24^c^	12.15 ± 0.34^b^	9.33 ± 0.61^c^	7.06 ± 0.22^d^
C20:4N6	0.59 ± 0.05^f^	0.66 ± 0.06^f^	0.71 ± 0.03^f^	60.70 ± 2.18^d^	81.90 ± 0.98^c^	96.90 ± 1.61^b^	106.8 ± 5.30^a^	60.37 ± 1.46^d^	37.27 ± 0.80^e^
C20:5N3	0.83 ± 0.09^d^	0.88 ± 0.08^d^	0.63 ± 0.45^d^	3.63 ± 0.17^b^	N	N	N	4.79 ± 0.12^a^	2.56 ± 0.15^c^
C22:6N3	1.83 ± 0.08^b^	1.46 ± 0.18^c^	1.42 ± 0.10^c^	3.10 ± 0.29^a^	N	N	N	3.28 ± 0.16^a^	3.35 ± 0.10^a^
**PUFAs**	**647.36 ± 5.17** ^**g**^	**618.84 ± 6.91** ^**g**^	**449.61 ± 3.07** ^**h**^	**3446.75 ± 14.36** ^**e**^	**4811.72 ± 151.4** ^**c**^	**5374.53 ± 69.72** ^**b**^	**6103.35 ± 33.24** ^**a**^	**3544.30 ± 58.41** ^**d**^	**2252.51 ± 60.03** ^**f**^
**UFAs**	**1199.89 ± 10.42** ^**g**^	**1116.97 ± 5.87** ^**g**^	**820.23 ± 6.56** ^**h**^	**9571.91 ± 50.11** ^**e**^	**13279.68 ± 94.73** ^**c**^	**14678.56 ± 212.6** ^**b**^	**17261.45 ± 199.4** ^**a**^	**9741.35 ± 155.7** ^**d**^	**6323.24 ± 61.94** ^**f**^
**FAs**	**1723.83 ± 9.77** ^**g**^	**1584.37 ± 6.34** ^**h**^	**1160.67 ± 7.96** ^**i**^	**10562.44 ± 48.76** ^**e**^	**14649.7 ± 95.55** ^**c**^	**16155.63 ± 198.1** ^**b**^	**18904.51 ± 200.4** ^**a**^	**10865.18 ± 153.3** ^**d**^	**7056.44 ± 60.71** ^**f**^

*Note*: Different lowercase letters in the same line indicate significant difference (*p* < .05); A: fresh rabbit meat; B: marinated for 10 min; C: marinated for 20 min; D: fried for 5 min; E: stir‐fried for 10 min; F: stir‐fried for 15 min; G: fried for 20 min; H: stir‐fried with spices; I: the finished product; N: undetected.

In stages A–C, the concentration of FAs decreased significantly (*p* < .05), which may be due to the degradation of fatty acids. In stages D–G, the concentrations of FAs increased significantly (*p* < .05), which may be due to the addition of canola oil. In stages H–I, the concentration of FAs decreased significantly again (*p* < .05); the downward trend was more obvious, probably because heating accelerated the degradation of fatty acids and the occurrence of thermal oxidation reactions. The concentrations of C16:0 (palmitic acid) and C18:0 (stearic acid) were higher than those of other SFAs. The concentration of C18:1N9C (oleic acid) was high; among PUFAs, the contents of C18:2N6C (linoleic acid) and C18:3N3 (linolenic acid) were the highest, which was highly consistent with the results of HS‐SPME‐GC/MS. Marcon et al. (2020) found that addition of appropriate amounts of curcumin reduced the concentration of stearic acid (C18:0) effectively in a study on fatty acids in mutton. By increasing the contents of oleic acid (C18:1N9C) and palmitoleic acid (C16:1), Mancinelli et al. (2021) showed that people who reduced their intake of SFAs and increased their intake of PUFAs, especially those of the N‐3 series of PUFAs, achieved a relatively balanced lipid structure.

### 3.5 Analysis of flavor substances during the processing of boneless cold‐eating rabbit meat

**FIGURE 2:**
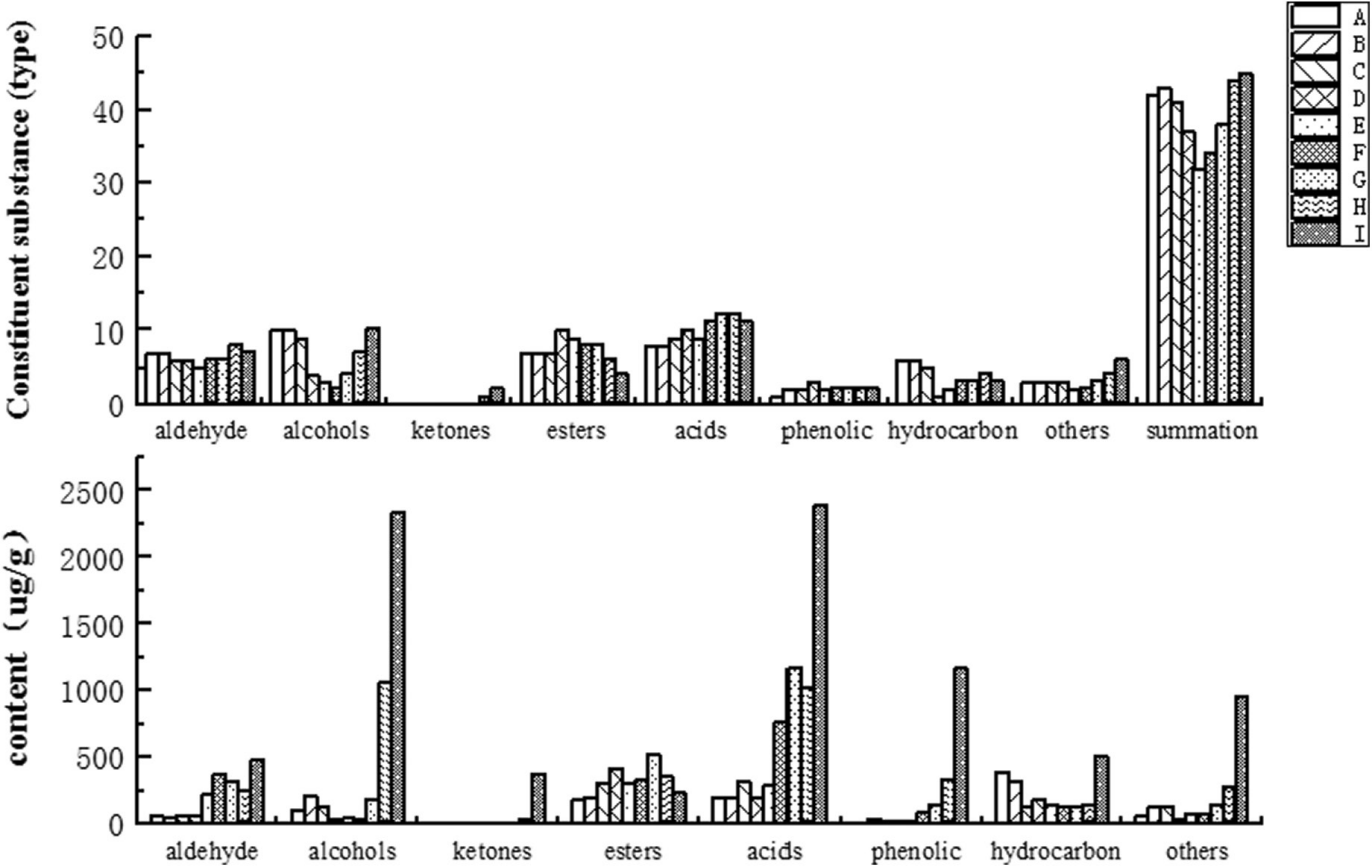
Types and contents of flavor substances in boneless, cold‐eating rabbits during processing.

In total, 77 volatile flavors were detected during the processing of boneless cold‐eating rabbit meat; 42, 43, 41, 37, 32, 34, 38, 44, and 45 volatile flavors were identified in the samples during stages A–I (Figure 2). Most flavor substances were generated in stage I. Esters and acid compounds were the main flavor substances produced during the entire processing. The total concentration of volatile flavor substances generated during stages A–E decreased gradually, while it increased sharply during stages F–I. Therefore, stage F can be considered the pivotal point for the production of volatile flavor substances. After stage F, concentrations of aldehydes, acids, phenols, hydrocarbons, and others increased significantly (*p* < .05), which might be due to the Maillard reaction or degradation of fatty acids, and the degree of oxidation increased with the time of frying. The concentrations and types of flavor substances reached their maximum by the finished product stage.

**FIGURE 3:**
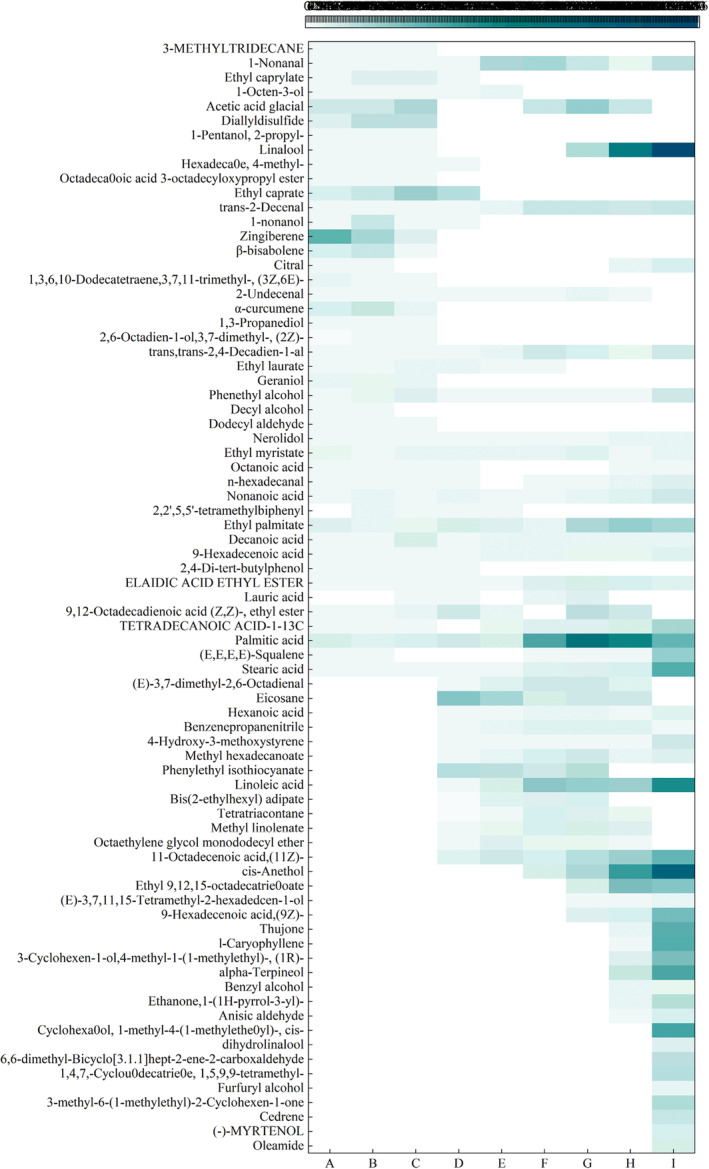
Heat map of volatile flavor compounds in boneless, cold‐eating rabbits during processing.

Alcohols, esters, and hydrocarbons are the main flavor substances produced during the entire process, which were transformed gradually into alcohols, acids, and others. The content of aldehydes first decreased and then increased during stages A–E (Figure 3). Aldehydes may be generated via some metabolic processes involving carbohydrates or the degradation of amino acids (Liu et al., 2019), which can impart floral, fruity, and strong oily flavors to boneless cold‐eating rabbit meat (Dirinck et al., 1997). The concentration of alcohols first decreased and then increased; alcohols can exhibit a special smell and spicy flavor. Ketones are produced via the oxidation and degradation of PUFAs. The concentrations of esters and acids first increased and then decreased. Esters, the products of the esterification reaction of acids and alcohols, have a fruity and sweet taste. Acidic compounds were produced via the hydrolysis and oxidation of fats (Lv et al., 2022). Phenols were derived mainly from the pyrolysis of cellulose and lignin (Mussen & Jianchun, 2009). Alkanes were formed via the homolysis of fatty acid alkoxy radicals.

## 4 CONCLUSION

The processing of cold‐eating rabbit meat followed the ATP → ADP → AMP → IMP→HxR → Hx → Xt pathway. 5′‐IMP exhibits the umami taste, and it worked in synergy with chloride to contribute to the umami taste as the main flavor in boneless cold‐eating rabbit meat. During the entire processing, the concentration of TFAA showed an obvious upward trend (*p* < .05). The concentration of Glu increased the most and it contributed significantly to the umami taste.

In total, 27 medium‐ and long‐chain fatty acids were detected, which included 13 SFAs, 6 MUFAs, and 8 PUFAs. Among the SFAs, C16:0 (palmitic acid) and C18:0 (stearic acid) were the most abundant, while C18:1N9C (oleic acid) had the highest concentration among MUFAs; among PUFAs, the contents of C18:2N6C (linoleic acid) and C18:3N3 (α‐linolenic acid) were the highest.

Seventy‐seven volatile flavors were detected, which mainly included alcohols, esters, and hydrocarbons that were transformed gradually into alcohols, acids, and others. Esters and acid compounds were the main flavor substances produced during the entire processing of cold‐eating rabbit meat. The types and concentrations of flavor substances changed before and after frying for 15 min. Therefore, the 15‐min stage of frying was the pivotal point for producing volatile flavor substances in boneless cold‐eating rabbit meat.

This study examined the correlation between common processes like marination and frying, and the taste of boneless cold‐eating rabbit. It also laid the groundwork for future research and development of cold‐eating meat products, aiming to provide a theoretical foundation for the industrial advancement of cold‐eating rabbit meat.

## AUTHOR CONTRIBUTIONS

**Xianling Yuan:** Conceptualization (supporting); data curation (lead); formal analysis (lead); funding acquisition (lead); investigation (lead); methodology (supporting); project administration (lead); resources (lead); software (supporting); supervision (supporting); validation (supporting); visualization (supporting); writing – original draft (supporting); writing – review and editing (lead). **Xianjie Peng:** Conceptualization (equal); data curation (equal); formal analysis (equal); funding acquisition (equal); investigation (equal); methodology (equal); project administration (equal); resources (equal); software (equal); supervision (equal); validation (equal); visualization (equal); writing – original draft (equal); writing – review and editing (equal). **Yidan Zheng:** Resources (supporting); software (supporting); supervision (supporting); validation (equal); visualization (equal); writing – original draft (equal); writing – review and editing (equal). **Yi Luo:** Data curation (equal); investigation (equal); methodology (equal); software (equal); validation (equal); writing – original draft (equal). **Hongbin Lin:** Formal analysis (equal); funding acquisition (supporting); investigation (supporting); methodology (supporting); project administration (supporting); validation (equal); visualization (equal); writing – original draft (equal). **Zhouyou Zhang:** Conceptualization (equal); data curation (equal); formal analysis (equal); resources (equal); validation (equal); writing – original draft (equal).

## FUNDING INFORMATION

The Key Technology of Industrialized Production of Sichuan Special Convenient Dishes [2020YFN0151].

## CONFLICT OF INTEREST STATEMENT

The authors declare no conflict of interest.

## ETHICS STATEMENT

This study does not involve any human or animal testing.

## INFORMED CONSENT

Written informed consent was obtained from all study participants.

## Data Availability

Research data are not shared.
